# Relationship between behavioral measures of anxiety and latent inhibition in mature rats

**DOI:** 10.3758/s13420-018-0331-4

**Published:** 2018-06-20

**Authors:** Elias Tsakanikos, Phil Reed

**Affiliations:** 1 Department of Psychology, University of Roehampton, Holybourne Avenue, London, SW15 4JD UK; 2 Department of Psychology, Swansea University, Singleton Park, Swansea, SA2 8PP UK

**Keywords:** Latent inhibition, Elevated plus maze, Anxiety, Schizophrenia, Rat

## Abstract

This study adopted a novel approach to relating nonhuman and human studies of anxiety and latent inhibition, by exploring the degree to which rats’ “temperaments” in relation to anxiety predicted the development of latent inhibition. It investigated whether anxiety levels in one situation (i.e., an elevated-plus maze) involving 38 intact, mature rats, could predict performance on a latent inhibition task (i.e., an animal model of attention), and, thus, reproduce findings from human studies. Rats were subjected to two tasks: a novel within-subject, appetitive stimulus pre-exposure procedure, and an elevated-plus maze task. In the stimulus pre-exposure task, non-reinforced exposure to a light led to facilitation of conditioning (perceptual learning) during the first 3 days, and to retardation of conditioning (latent inhibition) during the last 5 days. In the elevated-plus maze task, moderate levels of anxiety were observed. Regression analyses revealed that anxiety levels (plus maze) were a significant predictor of latent inhibition (stimulus pre-exposure). Measures of locomotor activity did not predict performance on the latent inhibition task. Rats with moderate levels of anxiety had better performance in the late inhibition task than animals with low levels of anxiety. These data and the methodology have implications for understanding nonhuman models of schizophrenia, and for the design of studies investigating these issues with nonhumans.

Latent inhibition refers to the phenomenon in which non-reinforced pre-exposure of a stimulus (CS_PE_) results in retardation of subsequent conditioning to this stimulus, compared to conditioning to a non-pre-exposed stimulus (CS_NPE_). This phenomenon has been taken to reflect some form of selective attention (Mackintosh 1975; Pearce & Hall, 1980; but see Bouton, 1993; Esobar, Oberling, & Miller, 2002; Reed, 1995, for alternative views) in that it corresponds to the ability to selectively ignore irrelevant stimuli (CS_PE_) and focus on potentially relevant novel stimulus (CS_NPE_). Attenuated levels latent inhibition (i.e., normal levels of conditioning following stimulus pre-exposure) in individuals with schizophrenia (Baruch, Hemsley, & Gray, 1988) and high psychometrically-defined schizotypy (Shrira & Tsakanikos, 2009) has been attributed to these individuals continuing to attend to the pre-exposed stimuli, maintaining attention to those cues, resulting in typical conditioning (e.g., Lubow, 1989; Lubow & Weiner, 2010).

However, it has been suggested that reduced attention to pre-exposed stimuli may also be a consequence of anxiety, rather than reflecting schizophrenia or schizotypy (Braunstein-Bercovitz, 2000; Braunstein-Bercovitz, Rammsayer, Gibbons, & Lubow, 2002). Although a link between anxiety and latent inhibition is not well established, there are a number of suggestive lines of evidence in support of this hypothesis (see Braunstein-Bercovitz et al., 2002). Given this, further evidence of such an anxiety-attention relationship would be of theoretical interest. For example, levels of latent inhibition are known to be impacted by dopamine (Gray, 1998; O’Callaghan, Bay-Richter, O’Tuathaigh, Heery, Waddington, & Moran, 2014; Weiner & Feldon, 1997), and both schizotypy (Caplan & Guthrie, 1994; Grant, Kuepper, Mueller, Wielpuetz, Mason, & Hennig, 2013) and anxiety (Peroutka, Price, Wilhoit, & Jones, 1998; Rebolledo-Solleiro, Araiza, Broccoli, Hansson, Rocha-Arrieta, Aguilar-Roblero, Crespo-Ramírez, Fuxe, & Mora, 2016) are associated with increased dopaminergic activity.

In line with these observations, experiments with humans employing both negative priming and Stroop tasks have shown that individuals with high levels of anxiety can show a lack of inhibition of attention to irrelevant stimuli (Berggren & Derakshan, 2013; Dalgleish, 1995; Eysenck, MacLeod, & Mathews, 1987; Fox, 1993; Van Den Hout, Tenney, Huygens, Merckelbach, & Kindt, 1995). Braunstein-Bercovitz (2003) investigated the effects of stress on negative priming by threatening a subject’s self-esteem, and noted that, as stress increased, negative priming diminished. Although the relationship between negative priming and latent inhibition is not straightforward, due to the multiple possible interpretations of latent inhibition (cf. Lubow, 1989; Mackintosh 1975; Pearce & Hall, 1980; Reed, 1995), such results are consistent with a view that reduced latent inhibition may be associated with anxiety, which often accompanies schizophrenia (Gorun, Cieslak, Harkavy-Friedman, Deptula, Goetz, Goetz, & Malaspina, 2015; Huppert, Weiss, Lim, Pratt, & Smith, 2001) and schizotypy (Braunstein-Bercovitz, 2000; Lewandowski, Barrantes-Vidal, Nelson-Gray, Clancy, Kepley, & Kwapil, 2006). In fact, Braunstein-Bercovitz (2000) noted in a multiple regression analysis that anxiety had a stronger impact on attenuation of latent inhibition than schizotypy. However, given the varied possible psychological mechanisms underpinning the latent inhibition effect LI effect in humans and nonhumans (Lubow, 1989; Mackintosh 1975; Pearce & Hall, 1980; Reed, 1995), evidence that this relationship holds in the conditioning procedures traditionally used to study these effects in nonhumans would be of use in the development of models and understanding of this process.

In fact, nonhuman studies have highlighted a relationship between anxiety and reduced latent inhibition. Stressed rats display increased dopamine activity (Mizoguchi, Yuzurihara, Ishige, Sasaki, Chui, & Tabira, 2000), which is known to reduce latent inhibition (Weiner & Feldon, 1997). Similarly, when corticosterone (a hormone secreted in response to stress) is injected into rats, then latent inhibition is disrupted (Shalev, Feldon, & Weiner, 1998). These results suggest that anxiety and stress (rather than schizophrenia) disrupt latent inhibition – a finding that has a direct implication for understanding models of schizophrenia based on paradigms such as latent inhibition.

Unfortunately, there are problems in making direct comparisons between the nonhuman and human studies, as nonhuman studies induce a stressor through an experimental manipulation (Lehmann, Stohr, & Feldon, 2000; Shalev et al., 1998), and human studies typically rely on measures of “temperament” or measuring levels of anxiety. There have been no studies examining the relationship of anxiety to latent inhibition in intact rats without an intervention to provoke stress. The existence of numerous possible problems in equating the response to a stressor in nonhumans to a long-standing anxiety in humans makes extrapolation from such nonhuman studies difficult. This gap in the literature makes problematic direct comparison between nonhuman studies that show a stressor negates levels of latent inhibition, and many human studies that have shown a negative relationship between anxiety and attenuated latent inhibition. In turn, this has some impact on the development of models of schizophrenia/anxiety that are based on nonhuman studies (see Lubow & Weiner, 2010).

The current study aimed to explore the relationship between anxiety and latent inhibition in in rats without using a stressor by employing techniques from the growing study of “behavioral types” or “behavioral syndromes” – exploring the within-subject similarities in performance across a number of tasks (Araya-Ajoy & Dingemanse, 2014; Byrom & Murphy, 2018; Sih, Bell, & Johnson, 2004). In doing so, this presents an opportunity to develop a novel approach to relating nonhuman and human studies of anxiety and latent inhibition, as in such an approach the rats’ stress levels are not manipulated, but the animals’ “temperaments” are used as the predictor for the development of latent inhibition, as they often are in studies using humans (Braunstein-Bercovitz, 2000).

To these ends, a within-subjects latent inhibition protocol using an appetitive conditioning paradigm was employed. In itself, the use of appetitive procedures for the study of LI is not novel (Reed, Anderson, & Foster, 1999), but there have been only few such investigations using a within-subject procedure, and the results of such investigations are, in themselves, of interest. Rats were initially exposed to a to-be-conditioned stimulus (CS_PE_), and then conditioned to CS_PE_ and CS_NPE_ (a non-pre-exposed stimulus). The rats' anxiety levels were assessed using the Elevated Plus Maze (EPM; Handley & Mithani 1984), which is a widely used ethological model for anxiety. The EPM consists of two open and two enclosed arms, with the two open arms and the two closed arms facing each other. The open arms are said to represent an anxiety-provoking environment (Pellow, Chopin, File, & Briley, 1985). An anxiogenic effect is defined as an increase in time spent in the closed arms, hence increasing the aversion of the anxiety-provoking open arms. In contrast, greater exploration of the open arms suggests a decrease in the natural aversion of the open arms, and an anxiolytic effect (Hogg, 1996). If rats show similar effects to humans, then latent inhibition should be negatively related to anxiety levels.

## Method

### Subjects

Thirty-eight male Lister Hooded rats were used as subjects. They were approximately 12 months old at the start of the study. The animals were housed in groups of four, with water constantly available in the home cage. The rats had a free-feeding body weight range of 460–600g, and were housed in groups of four, with water constantly available in the home cage. The rats were maintained at 85% of their free-feeding weight throughout the experiment. All animals were weighed every day, and they were separated from the group, and housed and fed individually, overnight, if their weight varied away from 85%.

### Apparatus

#### Conditioning chambers

Training was conducted in four identical operant-conditioning chambers (Camden Instruments Ltd.), from which the levers had been withdrawn. The chambers were ventilated by a fan that also provided a 68-dB(A) background noise. The reinforcement, one 45-mg food pellet, was delivered to a food tray, which was covered by a clear, Perspex® hinged-flap. A micro-switch was operated when the flap was opened. A jeweled house-light was located on the center of the chamber ceiling (overhead light). Another light was located centrally on the chamber wall above the food tray (central light). Both lights were 2.8 W bulbs. Based on past studies (Reed et al., 1999), both stimuli were of equal salience.

#### Elevated plus maze

**Fig. 1:**
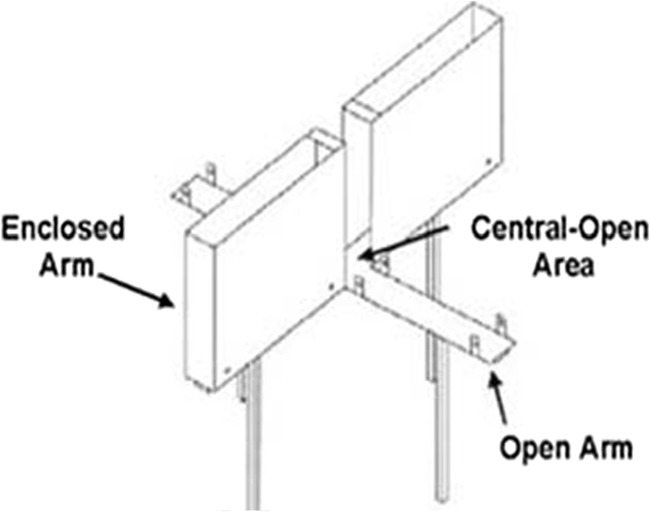
Schematic representation of an elevated-plus maze (from Augusta University; http://www.augusta.edu/core/labs/sabc/elevatedplusmaze.php)

The EPM consisted of two open (35 cm × 12 cm) and two enclosed arms (35 cm × 12 cm × 40 cm) and a center square (12 cm × 12 cm). The maze was elevated 50 cm above the floor. Open arms were surrounded by a 0.5-cm ledge and the entire floor was covered in black rubber. A black surround was placed around the apparatus to minimize visual cues. A schematic representation of an EPM is shown in Fig. 1.

### Procedure

#### Stimulus pre-exposure task

Phase 1 (pre-exposure) consisted of eight 30-min sessions. In each session, the subjects received ten 30-s non-reinforced exposures to a light (CS_PE_). For half of the subjects, the central light was used as the CS_NPE_, whilst the overhead light was used as the CS_PE_. For the other half, the central light was used as the CS_PE_, whilst the overhead light was used as the CS_NPE_. The first stimulus presentation occurred 150 s after the onset of the session. All subsequent inter-trial intervals were 150 s.

Phase 2 (conditioning) consisted of six 30-min sessions, during which all subjects received ten 30-s presentations of the CS_PE_, immediately followed by reinforcement. In addition, they received ten 30-s presentations of the stimulus CS_NPE_ immediately followed by reinforcement. The presentation of the experimental events was counterbalanced using a random, computer-generated order. Responses were recorded as entries to the magazine flap. All the subjects received the same programmed events.

#### Elevated plus maze

On the day of testing, subjects were removed from their home cages and transported individually to the testing room. Each subject was placed in the center square facing an open arm, and was allowed to explore freely the apparatus for a period of 5 min. Each 5-min trial was videotaped, and later analyzed by a trained observer using specifically designed software (Mazetime, Oxford, UK). The analysis of rats’ behavior in the maze was undertaken “blind” to the rats’ performance after both behavioral tests were completed. The EPM was cleaned with a 20% ethanol solution between trials (Bulos, Pobbe, & Zangrossi, 2015).

A variety of behavioral measures were recorded that have been shown to selectively reflect anxiety and locomotor activity (Cruz, Frei, & Graeff, 1994; Pellow et al., 1985). Anxiety parameters are taken to be reflected in the rats’ preference for the closed sections of the maze as opposed to the open sections (Cruz et al., 1994; Pellow et al., 1985). These consist of: (a) the number of entries made by subjects into the open arms relative to overall arm entries (ratio of open-entries, ROE – i.e., number of open-arm entries/total arm-entries); and (b) the time spent by subjects on the open arms relative to overall trial duration (ratio of open-time, ROT – i.e., time spent in open-arms/overall trial duration [300 s]). Low values for these anxiety measures indicate higher anxiety, and higher values indicate lower anxiety (same preference for closed and open places). The number of entries made into closed arms relative to overall entries (ratio of closed-entries, RCE), and the time spent by subjects in the closed arms relative to overall trial duration (ratio of closed-time, RCT), were recorded as well. The locomotor measures consisted of the total number of entries into any of the maze arms (total-entries, TE), and the number of entries into the closed arms (closed-entries, CE). The subject’s location on the maze was defined as four paws being present in a maze arm. These estimate the overall activity of the rat rather than any preference for particular areas of the maze – and reflect the degree of activity of the rat independent of anxiety.

## Results

**Fig. 2:**
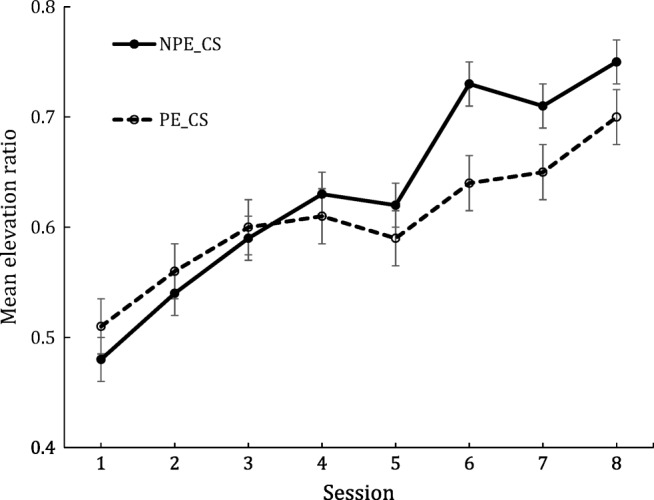
Mean elevation ratio in eight sessions for the pre-exposed (PE_CS) and the non-pre-exposed stimulus (NPE_CS). Error bars = standard error

Figure 2 shows the mean elevation ratios for the eight conditioning sessions. The elevation ratio was calculated by measuring the total number of magazine entries made during a CS period, and dividing this number by the sum of the entries made during a CS period and the entries made during the 30 s prior to a CS. Inspection of Fig. 1 shows a gradual increase of elevation ratio to both stimuli across the sessions. In the first three conditioning sessions, the elevation ratio to the non-pre-exposed stimulus was constantly numerically lower than that to the pre-exposed stimulus. After the third session, however, these ratios reversed so that the mean elevation ratio was constantly lower for the pre-exposed stimulus, and this pattern of results remained for the rest of training.

Given that such repeated-measures data often violate assumptions regarding sphericity, a Greenhouse and Geisser adjustment to degrees of freedom was adopted for this, and all subsequent analyses, as suggested by Howell (1992, p. 446). A two-way, repeated-measures analysis of variance (ANOVA) with stimulus type (pre-exposed vs. non-pre-exposed) and session of conditioning as factors was conducted on these data. This ANOVA showed the main effect of stimulus type not to be significant, *F*(1,38) = 2.40, *p* > .10, *η*^*2*^_*p* = ._059 [95% CI = .000– .278], but there was a significant effect of session, *F*(4.1,155.4) = 29.40, *p* < .001, *η*^*2*^_*p*_ = .431 [.310–.519], and a significant interaction between stimulus type and session, *F*(5.4, 207.5) = 4.10, p < .001, *η*^*2*^_*p*_ = .090 [.018–.157]. To investigate the significant interaction, simple effect analyses were conducted between the stimuli on each of the conditioning sessions. These analyses revealed no significant difference between the stimuli over the first five sessions, all *F*s < 1, but, on Sessions six–eight, inclusive, the non-pre-exposed stimulus had a higher elevation ratio than the pre-exposed stimulus, smallest *F*(1,207.5) = 3.97, *p* < .05 *η*^*2*^_*p*_ = .019 [.000–.070].

Table 1 revealed that subjects displayed a preference for the unprotected areas of the EPM, with higher scores for ratio-open entries as compared to the ratio of closed-entries, and higher ratio of open-time as compared to the ratio of closed-entries and ratio of center-time. Additionally, inspection of the means for ratio of open-entries and ratio of open-time (.57 and .47) suggests, overall, moderate levels of anxiety. Individual differentiation in term of responses was evident, as anxiety measures ranged from .33 to 1, and from .16 to .95.

**Table 1:** Descriptive statistics for the elevated-plus maze measures

	Mean	SD	Min.	Max.
ROE	.57	.12	.33	1.00
ROT	.47	.18	.16	.95
RCE	.42	.12	.00	.67
RCT	.35	.12	.00	.62
CE	5.31	1.91	.00	8.00
TE	12.23	2.48	5.00	17.00

*ROE* ratio of open-entries, *ROT* ratio of open-time, *RCE* ratio of closed-entries, *RCT* ratio of closed-time, *CE* closed-entries, *TE* total-entries

A series of correlations confirmed that the measures reflected separately anxiety and locomotor behavior. Table 2 presents the correlation matrix (Pearson’s *r*) for the two measures of anxiety (ROE and ROT), and the two measures of locomotor activity (TE and CE). The correlation between ROE and ROT was significant. The correlation between TE and CE was also significant, as the latter measure (closed-entries) is incorporated in the former (total-entries). Both ratio of open-entries and ratio of open-time, as well as closed-entries and total-entries, were positively correlated to each other, in line with previous factor-analyses showing that they detect anxiety and general activity, respectively (see Cruz et al., 1994, for a review). Furthermore, inspection of Table 2 shows that the relationship between anxiety and locomotor measures was found to be negative in all cases, replicating similar patterns of results from past studies that had employed the EPM (see Cruz et al., 1994, for a review).

**Table 2:** Correlations between anxiety and locomotor activity measures

	ROT	CE	TE
ROE	.739*	-.885*	-.462*
ROT		-.636*	-.313
CE			.771*

*ROE* ratio of open-entries (anxiety measure 1), *ROT* ratio of open-time (anxiety measure 2), *CE* closed-entries (locomotor activity measure 1), *TE* total-entries (locomotor activity measure 2)

p* <. 001

Mean elevation ratios to the pre-exposed CS (CS_PE_) and mean elevation ratios to the non-pre-exposed CS (CS_NPE_) were used to extract a latent inhibition score (CS_NPE_ minus CS_PE_) from sessions in which conditioning to the CS_PE_ was maintained constantly lower than to CS_NPE_. These scores were collapsed across sessions to form an average. Multiple regression analysis was used to determine whether anxiety scores (both separately and combined for the two anxiety measures) could predict the amount of latent inhibition (LI scores).

Table 3 presents a summary of the analysis; the first anxiety measure (ROE) significantly accounted for the 15.8% of the total amount of variance of the latent inhibition scores. The relationship between these two variables was negative. The second measure of anxiety (ROT) accounted for the 11.6% of the variance of latent inhibition, and the relation between the two variables was negative. The combination of the two predictor-variables (ROT + ROE) accounted for 16.3% of the total amount of variance of the latent inhibition scores. Similar analysis for the locomotor activity measures showed that neither of the two measures (closed arm entries – total entries), nor their combination, was a significant predictor of latent inhibition (all *F*s < 1).

**Table 3:** Regression analysis for the LI scores^a^

	ROE	ROT	ROE + ROT
_r_2	.158	.116	.163
**F**	6.77*	4.73*	3.41*
df	1, 36	1, 36	2, 35

^a^The difference between mean elevation ratio to pre-exposed CS and mean elevation ratio to the non-pre-exposed CS (LI scores) is the dependent variable; anxiety measure 1 (ROE, ratio of open entries) and anxiety measure 2 (ROT, ratio of open time) are the independent variable

*p < .05

## Discussion

The study sought to investigate whether behavioral measures of anxiety in rats would be associated with performance in a latent inhibition task. This was a novel approach to the study of the relationship between anxiety and attention in rats, as it did not rely on inducing stress in the rats, but measured their temperamental dispositions to anxiety, as is often done for humans (Braunstein-Bercovitz, 2000). In terms of the rats’ anxiety, it was shown that both ratio of open-arm time and ratio of open-arm entries in the EPM were associated with the amount of obtained latent inhibition. This pattern of results provides some support for the notion that anxiety modulates the development of latent inhibition (Braunstein-Bercovitz, 2000), and generally supports the idea that latent inhibition is modulated by anxiety, as has been shown so far in human experiments using very different approaches to its measurement (Berggren & Derakshan, 2013; Braunstein-Bercovitz, 2003; Van Den Hout et al., 1995).

These results are intriguing on a number of levels and have theoretical and methodological implications for this area. In particular, they imply that the use of latent inhibition as a model for schizophrenia/attentional disorders in rats will need some further investigation. In most models that use such a procedure, no account is taken of additional “temperamental” traits of the rats, such as their level of anxiety (see Gray, 1998; Lubow & Weiner, 2010), as it is in many experimental investigations employing humans (Tsakanikos & Reed, 2003; Tsakanikos, Sverdrup-Thygenson, & Reed, 2003). The current findings suggest the addition of measurements of a range of behavioral traits (Araya-Ajoy & Dingemanse, 2014; Sih, Bell, & Johnson, 2004) in the nonhuman subjects used in such studies might be needed to isolate the effects of anxiety or schizophrenia-related manipulations on attention (latent inhibition).

There are a number of procedural aspects of the current study that are worth mentioning for their implications for the investigation of this area. The current experiment presents a novel approach to investigating the relationship between human and nonhuman experiments relating to schizophrenia, attention, and anxiety because the rats’ stress levels were not manipulated for the purposes of the study, but rather the animals’ “temperaments” were used to predictor the development of latent inhibition. This approach is gaining currency across a range of investigations (Araya-Ajoy & Dingemanse, 2014; Byrom & Murphy, 2018; Sih et al., 2004), and adds a range of relevant dimensions to the discussion of how individual differences may affect learning processes, in ways that are obscured by the use of central tendencies (see Matzel, Wass, & Kolata, 2011). In addition, the current data also show the possibility of using a within-subject procedure to study latent inhibition in rats, which might have uses in the investigation of the impact of manipulations on that phenomena when the manipulations in themselves may be subject to large between-subject variation.

There are a number of issues that do deserve comment, and they may limit or shape the interpretation of these results. The results confirm that a latent inhibition effect was induced, but this only manifested during the later conditioning sessions. In the initial sessions, the opposite pattern was observed (i.e., perceptual learning), at least numerically, with responding to the pre-exposed stimulus being higher than that to the non-pre-exposed stimulus. Given that such an appetitive within-subjects design has never been employed before, a plausible explanation would be that in the first conditioning sessions the presentation of a CS_NPE_ (a novel, surprising event) elicited more orienting responses. This might have initially reduced the number of head intrusions into the food magazine in the presence of the CS_NPE_, resulting in a lower mean elevation ratio over the first few sessions for the CS_NPE_ than for the CS_PE._ When the orienting response habituated with extended training, latent inhibition was demonstrated. This suggestion will need to be explored in further experiments. However, it did make the latent inhibition effect, overall, quite small, and this might have impacted on its correlation with anxiety measures. Developing the procedure may help to further explore the precise nature of the attention-anxiety relationship.

The presumed attention-anxiety relationship noted in the current study was observed between a latent inhibition task and a behavioral model of anxiety suing the EPM. The use of only one measure of anxiety might be a limitation, and the work would be strengthened by showing a similar relationship with different measures of anxiety, such as the novelty suppressed feeding test, or with an aversive preparation, considering that the predictor used in the model is anxiety. It should be acknowledged the current study assumes that latent inhibition can be used as a measure of attention. Although many theories support this assumed link (e.g., Mackintosh 1975; Pearce & Hall, 1980), there are other views that do not assume that latent inhibition is an attention-based effect (e.g., Bouton, 1993; Esobar et al., 2002), and that the relationships between nonhuman and human studies in this area is not as solid as assumed (Byrom, Msetfi, & Murphy, 2018). Given that there is such a debate, an extension of the current findings using additional measures of attention would be useful.

The sample size used also imposes some limitations on the interpretation of these findings. These relate, in part, to the power of the tests employed. Although the fact that significant results were obtained with generally strong effect sizes mitigates some of these concerns, the lack of power may have meant that the “perceptual learning” effect noted early in conditioning in the LI task might have been significant with more participants. These limitations also relate to conducting further analyses with these data. For example, was the initial “perceptual learning” effect (or indeed the later LI effect) consistent across subjects, or did it also correlate with their anxiety scores. An analysis separating subjects into “higher anxiety” and “lower anxiety” may provide an answer to this question, but the sample size meant that any such analyses would not have sufficient power to be reliable.

In summary, the current results show that rats’ performance on a test commonly taken to measure attention (latent inhibition) is related to their performance on a task commonly taken to measure anxiety (EPM). This is a similar finding to many in the human literature using widely different procedures, and suggests that results from latent inhibition studies conducted in nonhumans may need to be reinterpreted, as far as they are related to schizophrenia, as such studies do not control for anxiety in their participants. Whatever the eventual outcome of such investigations, the current study also develops a novel procedure for simultaneously assessing the tendencies of nonhumans to display traits across a range of dimensions without the need for invasive procedures.
